# Reminiscences of Dr. Bonwill

**Published:** 1899-11

**Authors:** Eugene S. Talbot

**Affiliations:** Chicago, Ill.


### REMINISCENCES OF DR. BONWILL.

BY EUGENE S. TALBOT, M.D., D.D.S., CHICAGO, ILL.

In the death of Dr. Bonwill the dental art, especially its mechanical department, has lost its most zealous worker. The dental profession never before possessed, and many generations will pass ere it will again possess, such a genius. Bonwill was a genius who could not only invent, but could simplify the principles of those already invented, so better results were more easily obtained. There is no more difficult problem in mechanics.

He was a decided enthusiast as to his profession. Every patient, no matter how poor, received his best services. Bonwill had no equal as an all-round operator. There are many dentists in the profession who are good gold operators, good amalgam or cement workers, good at regulating teeth, etc., but Bonwill performed every variety of dental operations perfectly.

It was my good fortune to be one of a committee of five (from the Section on Stomatology of the American Medical Association) appointed at the Philadelphia session in 1897 to visit his clinic on producing anæsthesia by rapid breathing. Owing, perhaps, to lack of time and material, anæsthesia was not employed. The committee, however, were repaid by a clinic it would be difficult to duplicate. The clinic was held at nine A.M. (the only hour suitable for the committee) on the day subsequent to its appointment. Dr. Bonwill had collected forty-eight patients ranging in age from fourteen to seventy-eight years. These patients represented the extensive field of his practice. They were from Delaware, New York City, and Philadelphia. Every operation except crown- and bridge-work was exhibited. "Pyorrhœa alveolaris" was conspicuous by its absence. Every mouth was clean and healthy. Artificial dentures were accurately adjusted and articulation was perfect. Teeth, including the anterior lower, were built up and contoured with amalgam fillings. Spaces, clean and smooth, were noticed between all the teeth, points for which Bonwill was famous. His patients loved and admired him, hence his perfect control over them. A patient with an uncleanly mouth (no matter at what age) he would send away, telling him not to return until his mouth had been thoroughly cleansed, saying, "then I shall be pleased to operate for you." He spent much time in teaching his patients prophylaxis of the teeth and gums. He had the rare satisfaction of being able to say that he did not know what it was to have a case of "pyorrhœa alveolaris" in his practice.

His ingenuity was not confined to his specialty. He invented many useful things for general use.

Although Bonwill was not in the true sense of the word a pathologist, his genius often led him to appreciate pathologic etiology. From this came his favorite theory that improper articulation and want of spaces between the teeth were the sole causes of "pyorrhœa alveolaris." His farsightedness often reached beyond existing limits of dental practice. He was not a believer in crown- and bridge-work, because of the irritation produced about the gum margin and requirement of one or two roots doing the work of more. In place of this he built out the crown with amalgam, thus leaving the gum margin free, and inserted an adjustable plate in the place of bridge-work. Greater familiarity with the etiology of interstitial gingivitis will justify this conclusion of Bonwill and consequent change of methods of operation. His powers of minute observation rivalled that of "Sherlock Holmes." On being introduced to a dentist, he would say, "Let me see your office," and thereby he would size up the dentists and his methods.

It was my pleasure to travel four months with Dr. Bonwill in Europe, whence came an excellent opportunity to study the man and his methods. From the day we left until the very hour of our parting at New York Dr. Bonwill was busy. On shipboard he was continually writing. To his fellow-travellers he seemed the busy man in the party. He held clinics in nearly every country of Europe. At Moscow six afternoons until after dark were devoted to clinics. The large room was packed to the door. Not one-tenth of those present could see a thing he was doing, but his presence seemed to attract those of the section. No one, not even Virchow or Lombroso, received greater attention at the Medical Congress than Bonwill. The ovation at the surgical banquet surpassed everything at the meeting. He was lifted from the floor and carried to his seat at the table upon the shoulders of his friends, both dental and medical. At Berlin, Leipzig, St. Petersburg, Stockholm, Bremen, Paris, and London he held most enthusiastically attended clinics. The large number present at these clinics and the presents he received are but slight evidences of the many friends he made while abroad.

